# Mycotoxins: Analytical Challenges

**Published:** 2011

**Authors:** Hassan Yazdanpanah

Mycotoxins are natural secondary fungal metabolites produced on agricultural commodities. Mould and mycotoxin contamination may occur before harvesting, between harvesting and drying, and during storage. FAO has estimated that approximately 25% of the world’s crops are contaminated by molds and affected by mycotoxins, and the estimated loss extends to billions of dollars. Since fungi have a widespread distribution in the environment, mycotoxins are considered to be one of the most important contaminants in foodstuffs and feedstuffs. More than 500 different mycotoxins are known, but only a few present considerable food safety hazards. *Aspergillus*, *Fusarium*, and *Penicillium*are the natural fungal flora associated with foods. The most important mycotoxins are aflatoxins, ochratoxin A, zearalenone, deoxynivalenol, fumonisins, and patulin. The common occurrence of mycotoxins in foodstuffs and feedstuffs poses a threat to the humans and animals health. Mycotoxins have acute toxic, mutagenic, carcinogenic, teratogenic, estrogenic, and immunotoxic effects in humans and animals. Despite occurrence of mycotoxin contamination of agricultural products in the developed countries, mycotoxin exposure have greatly been reduced in these populations due to the presence of a legislatively regulated food processing and marketing system and the application of different strategies. However, in the developingcountries, much of the population relies on subsistence farming or on unregulated local markets. Therefore, regulatory limits for major mycotoxin classes and selected individual mycotoxins have been established in many countries to protect the consumer from the harmful effects of these toxins. The requirement to apply these regulatory limits has prompted development of analytical methods for the identification and quantification of mycotoxins in different foodstuffs and feedstuffs. 

The techniques used for detecting known mycotoxins are quite advanced and range from methods for directly detecting the toxins themselves, to methods for indirectly detecting the toxins. Modern technologies used for the detection of mycotoxins include mass spectrometry-based assays, ambient ionization mass spectrometry, electrochemical immunoassays, piezoelectric sensors, enzyme inhibition assays, biosensor arrays, and fluorescence polarization immunoassays. However, it is impossible to use one single method for analysis of mycotoxins due to the variety of chemical structures. Therefore, many analytical methods have been developed and validated. The demand for a fast, simultaneous and accurate determination of multiple mycotoxins, along with the heterogeneity of food matrices, creates extreme challenges for routine analysis. For simultaneous identification and determination of multiple mycotoxins, highly sophisticated multianalyte methods based on liquid chromatography coupled with multiple-stage mass spectrometry have been developed. Rapid analytical techniques such as infrared spectroscopy, fluorescence polarisation immunoassay, and optical biosensors, could be useful alternatives for screening and cost-effective quantitative determination of mycotoxins in the future. Rapid tests such as ELISAs, dipstick assays and lateral flow devices will continue to play a major role in routine analysis and for on-line applications. Molecularly imprinted polymers, evanescent wave technology, and microarray technology are among other emerging technologies for mycotoxin analysis. 

Although enormous progress has been achieved in mycotoxin analysis, there are still major challenges in this regard. Food is not necessarily safe, just because of the ruling out of the presenceof well-known mycotoxins. They might still be there in conjugated form, including masked or bound mycotoxins. Sampling is the largest source of variability associated with the mycotoxin analysis and the most crucial step in obtaining reliable results. 

In conclusion, although methods used for the detection of mycotoxins are quite advanced, the need remains for the exploration of novel detection technologies, for comprehensive validation of such technologies and for methods with increased throughput. Emphasis should also be laid towards the development of newer low-cost instruments for mycotoxin detection, which are reliable, portable, and easy to handle at field levels.

*Hassan Yazdanpanah is currently working as an associate professor of Toxicology, at the Department of Pharmacology and Toxicology, School of Pharmacy, ShaheedBeheshti University of Medical Sciences, Tehran, Iran. He could be reached at the following e-mail address: Hassan.yazdanpanah@gmail.com*

